# Supplementary material for: The psychological burden of NMOSD – a mixed method study of patients and caregivers
Source: PLoS One. 2024 Mar 29;19(3):e0300777. doi: 10.1371/journal.pone.0300777 (PMC10980246; doi:10.1371/journal.pone.0300777)
Supplement: S3 Data — (DOCX) [file pone.0300777.s003.docx]

Patient Focus Group Qualitative Data

| Vision | - Going blind was v. painful, like an icepick chipping at my brain. Went to a blind school to adapt. Lost my job – they don’t see me for who I am; they see me as a blind lady. - First few years of going blind was v. hard on me, anger, loss, cognitive stuff, complex migraine…got paranoid and wasn’t in my right mind”; “I transformed anger into advocacy”. - Vision blurry; can see a little light; on “bad eye days” less light, hard to focus, pain; black spots & fuzzy spots – sometimes they’re peripheral, sometimes right in front where I need to see. - Colors started to go wonky, extreme pain; When I blink I see movement - “I’m 28, went blind 13 years ago at age 15”; “I’m blind and I’m only 35”; “I’m 80% blind in right eye- it was my first symptom & how I got diagnosed” - Stressed about if/when will lose vision: “I’ve never had vision issues but I’m worried about what would happen if I got them…like how I’d be able to drive, how I’d interact with my kids” - Hot tubs, gym, shower, doing dishes, fevers: heat leads to vision loss - “If I do too much my eyesight gets worse and I have to ask other people to drive me around” - Reliance on assistive technology – but they conflict with each other - Doctors say I’m lucky as my vision loss is “mild”, but really my vision is blurry, “jiggling” – not just vision loss but vision impairments, doctors only focus on vision loss; the glare is significant – don’t do well in super bright environments |
| --- | --- |
| Pain | - Cramps, shooting pain, electric shocks down my leg/through my body. “Banding” and sunburn feeling: can only stand up at 10 min intervals; hurts to wear pants, regardless of the material. I wear shorts in the winter; sunburn feeling around chest, worse when I’m tired, can’t even have my hair brush against my chest. “It feels like a thousand fingers are pressing on my skin” - “Pain permeates every aspect of life”; “stress can be a trigger” - Months’ waits for appt with no skills to help cope in the meantime - No one asks re. how you’re coping emotionally with pain. “Painsomnia” Only get 3 hours sleep/night - Can’t really be outside in the sun – can’t drive in convertible with the roof down, sun really aggravates nerve pain |
| Paralysis | - Right leg was numb & couldn’t feel floor - Can’t feel fingers - Fell into a wall, semi paralysed from breastline down. Had a diagnosis (AQP4+ive) a few weeks later. “I shouldn’t complain…it could be worse” - Being paralyzed led to symptoms of PTSD |
| Impact on Physical Capabilities | - Walking affected substantially since latest relapse; need cane/walker; always in a wheelchair; balance issues; hard to carry things upstairs b/c need to hold railing - Have to wait a minute for cramps to settle down before can walk - “Walking a very big problem, I’m totally dependent on family members for activities and social life” - Used to go with family to trips, no casual trips now - only really important ones; can’t walk or hike long distances like I used to - Can connect with people who have similar conditions, understand my feelings, can play chess online more - NMO got me my job of teaching braille to children, can relate to them - Spouse insists on driving me everywhere, even to work. Spouse drives me at night as I can’t see to drive in dark. - I’m a yoga teacher and can barely do my own practice, because it triggers muscle spasms. - I still go to the gym 5 days/week- trying to stay very active through workout classes and weight-lifting |
| Sleep | - Either way too much or none at all; pulls all-nighters and then crashes - Pain meds makes me very “wired”, I schedule mid-day naps - Having a routine can be helpful; Acupuncture for sleep has been very helpful - “Sleep has been my top struggle since getting NMO “ - Anxiety about trying to sleep somewhere new if I go on vacation - Frequently using the bathroom during the night is disruptive - Diagnosed w/narcolepsy as a result of NMO – “I becomes tired at inappropriate times of the day” |
| Bowel & Bladder Function | - Can’t even have fun with wife/friends in outside world; so difficult and disheartening; Initially couldn’t even go out of the house; have to stay in the house until it takes its course - Always look for restroom in public; I’m so uncomfortable in public; Planning life around it more than would ever want to - I was discharged from hospital with a catheter bag for several months, now very hesitant of any side effects of new drugs that involve difficulty controlling urination and I won’t take them if so - Can’t walk, I have a full catheter & constipation - Prune juice, exercise, water, tried everything - I don’t want to explain bowel issues to new friends, so just don’t go |
| Anxiety/Worry about Relapse | - Relapses have gotten more frequent, to 6/year—spasms, discomfort in legs; I worry about having a relapse because I’ve had so many. “Goes hand in hand with planning for the future” - Hypervigilance to potential body symptoms: “Even hiccups can be a sign of relapse, and will scare the crap out of me”; “living in your head and constantly listening to your body”-can be very draining; “always wondering if any slight symptom is NMO”; any headache or body tension leads to the mental burden of “is it gonna come for me again?” - “I’m not suicidal but I can’t imagine living with this disease. I’m blind, it’s scary for me to think of the future. I’m out of treatment options, and I don’t know what will happen if I have another relapse. I’m only 35.” - I try hard not to think about what it would be like; “I try to keep fear of relapse on the back burner” - Worries about relapse contribute to insomnia and panic - “I don’t know how long I’ll have with my daughter” - When I start to feel symptomatic I’m very private about it but I’m internally panicking- leads to me feeling depressed; I keep things private, I’ll only tell my husband after 1-2 days go by, and when it’s almost a point of needing to go to the hospital - PTSD from attacks and hospitalizations. I’ve had therapy and was diagnosed with PTSD and it got to the point where going to the dentist to get numbed would lead to ptsd symptoms because I was previously paralyzed; Anyone who has been blind or paralyzed is terrified and has PTSD - I wish I’d addressed the high level of anxiety around relapse when I was first diagnosed - Worry about how loved ones handle relapse/symptoms |
| Sexual Dysfunction | - “People don’t talk as much about how sex life can be affected. “ - Reduced significantly because of uncontrollable bladder/bowel problems; “I can’t feel a thing, nothing down there works right, I have to take meds so I don’t pee myself.” - “I have cramps and my legs don’t want to work, so I can’t perform certain sexual acts”; “Don’t even know how to start, go about it, can’t walk, diminished sex life substantially”; Often doesn’t feel well, stomach issues, not in “the mood”; Pain with sex, muscle tightness, no libido - “Providers not able to help with decreased libido – “wait and see” is their response; Since the attack, I have no libido and no one can tell me what to do about it” - Chronic UTIs, can be peeing blood, throwing up because pain is so bad – impacts ability to have sex; leads to a lot of anxiety around sex, “I’d rather feel good and know I’d be fine than risk having a UTI” - Chronic yeast infections– went on for two years: “I’d rather not have sex to avoid a yeast infection” - It should be pleasure, meeting a basic need, should not feel terrible; results in lots of fatigue |
| COVID Pandemic | - Can’t go anywhere without a mask, have to be extra careful; extra isolated b/c of having a chronic health condition; “Others are living like it’s over and we can’t” - Hard b/c I’m so vulnerable. Son & partner want to live life as usual as they’re vaccinated. I’m constantly having to ask “you’re not sick, are you?“ when my son wants to come by. But I don’t always want to be harping on it. I want to have a normal relationship & not put parameters on it. - Worried that potential nerve damage from contracting COVID would exacerbate NMO; Contracting COVID made things worse with NMO brainfog, made it harder to learn; “COVID led to me having a flare”; increased frequency of muscle spasms; had an attack right after getting COVID - Fevers, UTIs, COVID are all triggers for existing damage to exacerbate - “The Pandemic saved my life” – I got in with a specialist v. quickly b/c no one was going to appointments. |
| Journey to Correct Diagnosis | - Originally misdiagnosed with MS, taking MS meds, forced into relapse, when go into Drs. offices they misdiagnose; Started in brainstem with nausea & vomiting – treated for MS, paralysed from waist down & then got the right diagnosis. Lost half vision in one eye. “You have to be your own advocate” to get correct diagnosis. - Being told it’s “all in your head” - Took a long time to get proper diagnosis, started dealing with this when 13—would get dots in eyes, couldn’t see, went to several eye doctors—all they told her was optic nerve atrophy—Finally got diagnosed with NMO 3 years later at age 16 - “Doctors failed at telling us how to live with this” - Applying liquid eyeliner, realized she couldn’t see & had eye pain, lost a lot of color perception. Went to the optometrist, who sent her right away to an emergency eye clinic (in Canada). During that time, when she would look down she’d get electric shock down her spine. She went for an MRI and they found long lesions on her spine. Stress from a pet’s death triggered another flair and pt went completely blind in a “white fog.” - Eye pain and twitching in arm, woke with no peripheral vision, next few days lost complete vision. Went to the ER they did an ultrasound on eye and didn’t see any damage. Then set her up with an ophthalmologist who didn’t see anything wrong with her eye. Then she was referred to an ophthalmologist-neurologist who diagnosed her with optical neuritis and sent her back to the ER and they put her on steroids and gave her an MRI. Had lesions on spin and in brain and optic nerve. Also had no balance or strength. Also did a lumbar puncture and was antibody APQ4 - Felt like his eyes were “off”, headache for a week, lost color vision and “everything was dark”. Went to ER, didn’t have health insurance, had to wait for specialist treatment due to prohibitive cost. He then got insurance, had first real attack after aunt died, leg was paralyzed and they ended up crashing car into neighbor’s mailbox. Then got a new doctor and got official diagnosis. Has had 13 active flares. - I started getting sick, no one knew what’s going on. Then I got paralyzed. I was going to cosmetology school, had to drop out. Then working in a hospital as a PCA, and started feeling nauseous. I was in full time school plus working, I thought I had a stomach virus. Then I went to the ER 2 or 3 times a week. Thought I was going to die. I was in the hospital for 5 weeks. I got fired from job for unexcused absence. 6 months later, I was sick again, and down for 4 months, prednisone helped, but then my hip started hurting. MRI showed I need a hip replacement from prednisone. I’m only 29 years old. |
| Employment | - Workplace discrimination: Made to read a license plate at a job interview - Having to constantly reiterate to people at work why I’m wearing a mask - “NMO has impacted my work a lot- I own a company and I can’t go to job sites and walk around…because of mobility and balance issues” |
| Future Plans | - Always wanted to go to college, have to keep self in healing at home and can’t go at this time; A lot of normal milestones hit at later times—graduate HS later, college later, will probably get married/have kids later; So much time spent on treatment planning and couldn’t focus on academics or other things as much; Lots of dreams and goals pushed back - The hardest part in the beginning when you have no clue what will happen next. The further from relapse, the calmer you get. It changes so many plans you had for your life: I can’t have kids, can’t plan a trip 2 years from now. Do I want to risk buying this house b/c what if I need an accessible house later? A constant planning in my head re. what ifs? - I’m trying to find a house to buy…what if something happens to me. My mom is my full-time aide. She doesn’t get paid if I’m in the hospital. So what if I buy the house & then can’t pay for it. - Difficulty planning for the future because reactions to tx can vary and symptoms can generally be variable - “It is what it is, I’m grateful that I’m not worse because I know I could be” |
| NMO as an Invisible Illness | - I want to turn my body inside out to show how much pain & electric shock & suffering I’m in; people can’t see shooting nerve pain like fire through my butt - I’m viewed as a high functioning person so lots get dumped on me, even by my doctors (e.g. writing advocacy letters); I’m “high functioning” and always keeping busy and running around, but then when I actually feel ill & fatigued, my family doesn’t get it - People who are immunosuppressed become an afterthought during the pandemic “why can’t you come out?” if they don’t see us in a wheelchair, they don’t realize we’re sick - I’m in a wheelchair – I have anxiety re. big crowds – I’m sitting lower, everyone’s above me, butts in my face. So many things aren’t accessible. Fell out of chair at football game. - I’ve been called out by cops and bystanders for having a handicap sign on my car as I look young & healthy - The illness varies day to day and it’s hard for people to believe that I can have days when I’m fine and days when I’m not; My adult son and husband don’t understand my body’s limitations; Some people “forget” I have NMO; People saying “you look great” even though I feel sick; “You don’t look sick” - Had access to transit within university to get from one class to another, driver said “she doesn’t have any mobility aids”; People don’t see the struggle when walking because I don’t have mobility aids |
| Impact on Family & Friends | - I’m high functioning and a good coper, so it’s an invisible illness for me. My adult son sticks his head in the sand about it. He was 17 when I was diagnosed. - Family and friends handled it really well, never treated me any different; Family very compassionate, supportive; Difficult for loved ones to see participant in pain; Adult children constantly offer to help. Husband always wants me to do less. - People commenting on weight gain/puffiness; Annoying comments like: “what’s wrong with you”, “oh I know someone who died of MS”; Having to continuously explain to people why I’m wearing a mask or why I’m being careful about hugging - NMO never being discussed with others - Invitations from friends disappearing, even for non-physical activities; I stopped partying and friends see that as me acting “better than them”; Hard to be young with this disease- feels very isolating. Having to choose friends who are into slower paced activities in life; lifelong friends from my teens have stopped hanging out with me because of my physical limitations. - Met new friends through new hobbies (e.g., swimming). Social abilities have shifted, some are good, and some aren’t as good. I have some friends who are willing to take slow walks around the neighborhood, but other friends have shown no effort and have completely distanced themselves from me because of the diagnosis |
| Impact of NMO on Children in your life? | - I’m only 28, want to have kids, afraid that could genetically pass NMO onto kids; I’m blind from this disease, is that gonna affect my ability as a parent? - My 10 year old daughter realized how sick I am. I feel like she has the weight of the world on her shoulders, like if she comes home from school with a cold and gets me sick…I feel so guilty about this. I try to distance NMO from my daughter. |
| Anything Else You’d Like Us to Know? | - Discrimination in workplace; stigmatization, people think you have COVID (when masking); diseased - I’ve gone to a psychologist & she doesn’t get it. She just cheers me on. It’s nice, but would be nicer to have someone who really understands the disease. - Don’t mean to be rude or mean, but it’s hard b/c I feel so bad all the time. I cherish good days. I never thought I’d have to say no to taking care of grandson. I want to know why this happened? I want to know why my own body turned on me when I had no prior illness. - Brainfog/memory—common among ppl with NMO; Contracting COVID made things worse with NMO brainfog, made it harder to learn - Doctors causing stress by being rude & making me advocate for myself, stress exacerbates my illness; Doctors not knowing what NMO is, or treating me like a “zoo animal” and like I’m on “display” because it’s a rare disease; My family doctor didn’t know much about it and I explained it to him - Stress re. juggling how much $$ I can keep in the bank & make with disability benefits. - NMO can make you prone to depression |

Caregiver Focus Group Qualitative Data

| Impact on your loved one’s physical health | - Daughter lost her vision completely and never returned, adjusting life to that - Banding—whenever stressed, it’s a trigger that makes the banding come back more aggressively - Legs can lock up at any minute (muscle spasms)—always mindful/alert of it (when pt is driving, walking); Sometimes her legs don’t work, arms don’t work, can’t feel her fingers, eyesight goes blurry. She’s independent but it’s tough when she can’t move her body; Daughter paralyzed, has a huge impact, she can’t gain employment, very frustrating for her - If we go anywhere, she needs to know where the bathroom is at all times, she has severe nerve damage from belly down - Had to change the house around and make it wheelchair accessible; Lots of concern at the beginning, needs to always prepare ahead of time for transportation because wife in wheelchair - “Their health is something we’re always aware of” - You have to become an advocate for that person’s accommodations - She doesn’t complain about symptoms because she doesn’t wanna burden me, so I have to be more alert to the symptoms/flags - I worry about pt being exposed to any/all potential viruses (flu, RSV, COVID) - Some good days, some days when body doesn’t work as well. - She can now move her arms but can’t feel her hands – completely limits her abilities (ex: can’t type, can’t use phone), everything has to be voice activated. She wants a job. She has a degree in mathematics, went to college, graduated with honors. Can’t even get a job as a tutor- “you’re blind, you can’t do it.” – hurts me, hurts her - When she became paralyzed, it was horrible. Became paralyzed in 3 days. Tough when the doctor walks in and asks her if she wants to be put on a ventilator. She was paralyzed from the neck down and could only turn her neck to the side, in so much pain. Doctor said I don’t think she is going to live. MS drug paralyzed her. - “Keeping her stress-free is *always* on my plate” |
| --- | --- |
| Impact on your loved one’s emotional health | - Lonely – I see people withdraw themselves from her bc they haven’t gotten a flu/COVID shot; “Having a rare disease is a lonely life”; She married at a young age which I don’t know would have happened if she didn’t have this illness, it wasn’t a good relationship - “In a dark place”; She went from being totally independent to totally dependent—stressful - As a mother, so hard to see loved one upset, in pain, NOTHING I can do to make it go away, very hard thing for a mother to deal with; Helplessness; Seeing her lose her bubbly personality - Couldn’t see Mom during pandemic—didn’t want to infect her (with COVID or other viruses) knowing what it could do—so hard to NOT see her during such a tough time - Terrifying to hear all of the different doctor’s different opinions; Bombarded by information immediately after diagnosis, overwhelming; Tough when diagnosed a long time ago, all the research/info out had terrible prognosis - Trying to keep her positive by shielding her from my own fears/negative thoughts; “We play a game of hide and seek, she hides what she goes through, and I hide what I goes through”; You can’t reveal any of your emotions, you have to be supportive - We cry, talk about it together, be honest with how we’re both feeling, acknowledge the struggle; I try to give her courage to be stronger - She’s a single mom, fiercely independent, we never know how much of the story she’s sharing with us—not knowing how she is coping causes anxiety/emotional strain for us - She tries to manage my feelings and worries about my emotional wellbeing - When it all started, our daughter wouldn’t go out in public outside of school. She got training in blind skills and she was able to figure it out over time. In the beginning, she was scared, almost depressed. We used to go sightseeing, but that didn’t exist anymore- we couldn’t go look at the animals at the zoo. We learned in time that was ok. Not being able to see the Christmas lights makes me sad. We can go to concerts. She had some great teachers. - I’m a lot more emotional than my daughter. She is very strong. We try to make everything she wants possible. She is very dependent physically but independent minded. - Worry that “One of these days we won’t be here” |
| Impact on your physical health | - I gets migraines due to stress… the body’s way of reminding me that this job is stressful |
| Invisible Illness | - She looks fine on the outside but I know she’s struggling so much; some days are good, some days are bad - “You get weird looks”, people don’t know that she has a vision problem, see the little mistakes she makes and thinks it’s odd (like walking in a puddle); I can read peoples’ facial expressions judging her, thinking “is she really blind?”; Had to stand up for wife several times as she has mobility issues, we have a handicap sign and people confront her about; “Have to justify to some asshole that she has a problem” - The kids don’t realize “why is mom going for treatment” when she seems fine - In-laws don’t understand/forget that she can’t travel and get upset—she appears fine on some days; Our inner circle not quite grasping NMO fully; friends don’t understand that she WANTS to hang out but CAN’T hang out; family doesn’t understand why pt can’t get into car and drive 2.5 hours for a family gathering—you’d think that family of all people would understand - People don’t recognize that she’s not dealing with the same level of immunity as you are - Symptoms of heat sensitivity, pain, mobility, muscle spasms, fatigue, neurological deficits and still has to wear diapers—most people think that he’s lazy, exaggerating things, and simply don’t know about NMO; “there’s a huge lack of compassion toward him because of the invisibility of his illness” - Have to explain to ER docs, bring them brochures, about what is NMO - She keeps things invisible from me! Doesn’t want me to worry, too proud to report symptoms sometimes |
| Worry about loved one having a relapse | - I life in constant fear of this, “it’s my biggest fear”; Always worry that a relapse will take us to the next level of impairment - I put cameras throughout the whole apartment to make sure she’s ok when I’m gone at work - Whenever pt’s back itches, I worry about it being a flare up; even minor symptoms make me intensely anxious about a potential flare up - We try not to think about it—we know that the possibility is always there—worry what that means for how we take care of our daughter—but for my own mental health we try not to think about it |
| How others’ view your role as caregiver | - Friends always used to say “isn’t she a burden to you??”; people say “aren’t you wasting your life away?” - They were married 10-12 years, she got diagnosed, her husband left because he decided “this is not what I signed up for”, now I’ve (Mom) stepped in to be her primary caregiver - “I don’t wanna be congratulated for taking care of someone who has taken care of me for my whole life” (daughter is caregiver for mom); I don’t want to be congratulated for things that any good child would do for a parent |
| The Pandemic | - Traveling, friendships, all restricted or entirely taken from her; Being in a wheelchair she worries if anyone is sick she would become infected and get sick so stays home - Everyday worried/concerned about kids going to school, going to work, missed a lot of time with kid/grandkid because wanted to keep distance as to not expose Pt; Have to be really careful with everything/everyone—“it’s like playing Russian Roulette” - Had to take pandemic so seriously—caused a lot of anger/resentment/stress toward people who were not being considerate; Terrified of COVID, fights with relatives/friends about COVID-denial - Pt had to be hospitalized when they got COVID (reported by many caregivers) - “Covid didn’t impact us because we don’t go anywhere” |
| Journey to correct diagnosis | - Originally diagnosed with MS, treatment made it worse, looking back “the whole time you were doing a harmful treatment plan”; Initially diagnosed with transverse myelitis (viral)—aqp4 test sent out and got results back but never was contacted by doctor, had another relapse - Symptoms showed up months prior to paralysis; tough that NMO wasn’t well known and more likely to get MS diagnosis; So many different neurologists, didn’t know what was going on, misdiagnosed with MS; Doctor suggested that she was having “female issues” and “hysterical” - Wife had first attack in 2011, she got to work & couldn’t see to log into her computer. Sent home. Massive headache, blurry vision. Opthamologist sent her to ER. Sent home w steroids. Lost sight in both eyes. Would go in for plasmapheresis for 2 weeks inpt, her sight would improve, then get worse 6 months later, do more plasmapheresis…..did this for 3 years. Then ended up w multiple blood clots & DVTs. Sent blood sample to Oxford. Saw prominent neurologist at Mayo, expert in NMO who told us “there’s no calories in prednisone” when she complained of weight gain. CG’s first anxiety attack b/c couldn’t find entrance to hospital at Mayo clinic..Total waste of time…demoralizing. - Some doctors said she would never recover, would die, you’d have to say goodbye to her - Every time they see a new doctor, the doctor always questions the diagnosis, have to “go through that whole process over again”; Lose a lot of faith/trust in doctors because have to re-explain everything every time they see a new doctor; Sending pt back and forth to hospital/doctors/specialists while feeling horrible (had to sit in hospital lobby for hours) and took so long to figure it out (1 year); Constant string of doctors- nausea, vomiting, vision loss overnight - When she contracted this disease, 1 doctor in 1000 knew about nmo, didn’t know how to treat it; Went 17 years with no treatment or the wrong treatment; Teenager when diagnosed, many doctor appts and didn’t know what was wrong with her. |
| 2 Topics added by loved ones | |
| Making changes in life to protect loved one | - Have to let friends/people go who won’t be COVID-safe and understand needing to be safe; Had to cut people off who don’t understand that she has to take care of self and patient - Can’t travel to home country for the safety of pt during COVID - We have a rough outline re. what we want to do this year – almost all of it is re. advocacy work. Anything longer than that, we don’t plan. Maybe a month in advance is as far as we go. “all plans are soft until an hour before” |
| Balancing caregiving with fostering independence | - She wants to be independent. We tried as best as we could. She got a guide dog at 16. We have had to let her make her own decisions. Found friends to help her get around; Hardest thing was turning over my daughter’s life to a dog. This dog got her to class, got her to the bus. We got her a cell phone so she could call for help. - I want to do things for her, but doing everything for her impedes her being independent. “If you help, I can’t learn to do that on my own.” – sometimes leads to arguments, but at the end of the day we love each other - Whatever she can do to get her independence (ex: we give her clothes but she puts them on, we do things that require more dexterity) |

Patient Interview Qualitative Data

| Pain and Nausea | - “So I have constant nausea… It's like having morning sickness all day long.” - “I have pain every single day and it's random, you know, electrical shots throughout my brain. It goes throughout my body. There's these muscle spasms that come and go. There's a lot of itching and just discomfort” - “Sometimes I get extreme pain in my back” - “It was like excruciating pain, kind of like waist down. It kind of just felt like your skin was like on fire.” - “The biggest thing I struggled with was pain… Like I was paralyzed, but pain [was worse than] paralysis because it was just so mentally hard [because] they couldn’t do anything for me. I was on a bunch of opioids, NSAIDs, SSRIs and nothing helped.” |
| --- | --- |
| Vision | - “Sometimes I’ll admit I get frustrated if I can't do something that I used to visually be able to do so easily. It takes so many more steps as a blind person to ‘complete one task” - I had to go to a blind school to get rehabilitation so that I’m able to get on a computer again. I can use a phone. I can cook. I can clean. I can be independent, but blindness is a very hard thing to adapt to if you're not born blind.” - “I was getting like dots in my eyes where it looked like I was just seeing black dots. I like it was weird. I'd be able to see, and then these black dots would show up, and I couldn't see for like a couple of days. Then I would come back to Normal, where I could see. So the first [symptom] was my eyesight loss” |
| Treatment/  Medical Care | - The first infusion I had I was terrified. I didn't know if this stuff was going to make me sick, which it did. I didn't know if I could handle it. I didn't know if I would have a reaction.” - “I didn't know what to do. My doctor basically told me, okay, now, you're blind. But he didn't give me any resources as far as, okay, so what do I do with this?” - “So I go get the referral, I go see the doctor, I’d come back with the advice, I’m sorry we can't do this because it's not FDA approved. You know, you get that whole song” - “When somebody develops a disability, the coping strategies there are really needed because even in training doctors, they just, they'll give you a diagnosis but then you're kind of left to deal with it on your own.” - “Doctors need to be trained on how to handle these serious medical conditions, and how best to get their patients to rehabilitation services. Or just to be compassionate and caring and just, you know, they can't wave a magic wand, and take it away, but at least to empathize a little bit more with the patient, instead of just that cold sterile, oh, you have this disease. Okay, well now what?” - “I had doctors tell me it’s all in my head when I was paralyzed… my respect for doctors has really gone down the drain…” - “I saw my [general practitioner] and said, ‘I’m walking around on legs that I can’t feel, something’s wrong,’ and she basically said to me, ‘Oh it’s a pinched nerve, lose ten pounds and you’ll feel better’”. - “In 2019 I got panic attacks for a good two months, and the doctors had to put me on prednisone and anti-panic medication, because the panic attacks would induce nausea.” |
| Employment/  Finances | - “I've been turned down for disability twice. The second time I was told that I needed to cut my work day back to six hours a day, and I said, I can't do that… I have to work seven and a half hours to have health insurance, and unless you're going to pay for this for me, I have to work my full forty hours, there’s no options on this.” - “The disease has really changed my life in a lot of …areas because I lost my job. I could no longer work. I couldn't drive.” - “I was one term away from being a medical assistant… and I ended up in the ER… I end up in the hospital a total of 5 weeks. The hospital fired me, saying I was on an unexcused leave of absence. And I’m like, I was in your hospital.” |
| Invisible Illness | - “Because I look normal, and because [sometimes] I might have a cane, it’s so strange because people walk up to me and they’ll go, ‘Oh what did you do your leg?’ …I get so tired of having to say, ‘there’s nothing wrong with my leg or my foot. I have NMO.’ And of course, even if I do say that, I get that look like, ‘what the heck is that?’ And I’ve gotten to the point where I just generalize it now, because everybody is going to say, ‘Oh, it’s like MS,’ and that used to make me so angry”. - “Sometimes I talk to my mom about the really bad days [I had in the past], and in her mind, those days are over because I’m not physically struggling with any [NMOSD symptoms] right now, at least to her. But for me, it’s really hard because it’s like I think about [NMOSD] every day, [NMOSD] isn’t over for me… Yesterday we were kind of talking about this, and she was saying how hard it was to go through that time [when I had my first relapse], and I’m like, well I still go through it.” |
| Sleep | - “I couldn’t sleep, I was having a great amount of pain” - “Fatigue is the symptom I struggle with the most. I just don't have energy throughout the whole day, all the time” |
| Anxiety | - “I was afraid to leave the house because I was afraid I’d fall” - “My husband and I made a trip to Virginia Beach back in September, and I worried the whole way down there. Can I do this?” - “There's a lot of questions and unknowns and that can create a lot of confusion. And you know, and just the feeling of, oh, my gosh, is something gonna happen? Is something going to happen tomorrow within the hours, so it just creates a lot of anxiety.” - “Any little pain. I'm like, okay, is it NMO? Is it just an infection like, okay? And if it's an infection, what do I do? And is it going to last forever? If it's an attack? Who do I go to? So it's just like literally, with any sort of ache and pain I get anxious.” - “I started getting really bad anxiety because I couldn’t see people… I got social anxiety, [wondering] what are people thinking about me not being able to see? And then [I} also can’t read facial cues, or you know, body language. So I got a bunch of anxiety from going blind… I couldn’t really participate in as much things as I used to.” - “When I feel like I'm getting blurry vision, and then I begin to feel be filled with fear.” - “I get panic attacks because I’m like I don't know which area is going to be affected next.” |
| Depression | - “I went through a whole year of grief. I couldn’t read anything online about NMO. I couldn’t do anything for about seven months after my diagnosis… [My doctors] wanted me to find a therapist, and I said, ‘You have to let me reconcile this in my head first, because I don’t even know what to say or do.’ I felt like I was standing on the edge of a cliff, ready to jump 24/7. My body felt like it was a rubber band stretched to the hundredth degree, and I was just waiting… waiting for another attack”. - “That anger… you know, you go through a lot of different grieving process of loss, because when you lose your job, you lose the ability to drive and be independent and function for yourself. You lose the ability to do things that were so easy to do.” - “I'm 23 years old. I should be out and having fun and living my life. I shouldn't have to be worrying about my health like this. …It's harder when, your peers are all healthy, so there can be anger and frustration when you start to not feel well.” - “So a lot of people [with NMOSD] gain a lot of weight um, which like, in addition to coping with a diagnosis you're now like. Oh, my God! My body looks different, and that's really really challenging.” - “I just turned 25, and you think 25 is the prime of life, and that everything is perfect, and you’re the hottest, and you know whatever… and everything changed. I went from being the kind of person who didn’t even take Tylenol to taking like 18 pills a day. I couldn’t see, I had trouble walking, and my entire life changed overnight… I’m so young, it just doesn’t seem fair” - “During the early stages of the diagnosis, I felt very pessimistic and unsure about the future, and I felt like it was like punishment. What’s the word for consequence of my past life, you know?” |
| Social Life | - “There's some family members that, you know, they just, they, they may not understand, and they may be disconnect because they don't know how to deal with it. Same with some of the friends that I’ve lost. They just, they don't know how to interact with me. And I haven't changed, I'm just blind. So, it's just different. But I’m still the same person.” - “I often do seek advice from other peers and, you know, people who have, you know, dealt with the disease for quite a while.” - “There are times where we can get frustrated with each other, and it's about communicating, and you know, and I try to do the best that I can. But sometimes you're in the heat of the moment and maybe you're not thinking as clearly, or sometimes the disease might prevent me from fully communicating my needs to him.” - “When my youngest son was about one year old, I couldn't use my right hand, and so I couldn't carry him, and it was so hard. It was so hard… he would be crying and wanting to be fed, and I can't carry him. And so I would just look at him and cry.” |
| Physical Quality of Life | - “I couldn't even feel the bottom of my feet, so I refused to drive. I said I, if I can't tell where I'm putting my feet. How am I going to know if I’m stopping, or going, or whatever, and I didn't drive for about four months before I got to where I felt comfortable doing it” - “My skin is desensitized in some areas and hypersensitive, and others, and on my feet toes there's only certain like there's little spots I can actually feel if somebody touches” - “You know it slows me down for sure. It made me throw away all my cute shoes, which made me mad” - “You know, the day my husband put a safety handle in the shower. I was mad.” |
| COVID/  Pandemic | - “So after I pretty much got over the actual virus [COVID], my body started freaking out, because, like you know, my immune system's all messed up. The symptoms I have were like my body was going numb, like from like shoulder down, just because the virus had wreaked, you know, havoc on my body for a bit. And then it just, my body just went numb, and it was not fun.” - “My husband and myself, like we haven't been able to go to any family functions and stuff, because I’m just so nervous of getting COVID. So that Thanksgiving, that first real holiday since the pandemic, wase the hardest - it was so sad. I just remember,me and my husband were sitting there. We made like a big dinner to try to make it feel normal. But even though it was just us, and it just like it was so sad. I just remember crying like this is not normal, right? Like it's not how it should be.” - “I feel it's like extra, because, you know, we're always worried about getting sick, and then it's like, well, not only do we have to worry about getting COVID sick, but it could make us go into a relapse, too. It's just so many extra things to consider.” - “Yeah, like we lived like total crazy people [during the beginning of the pandemic].” - “[The pandemic affected me] emotionally, especially in the early days of COVID. There was a lot of anxiety around it, like with all the warnings around people that were immunocompromised.”’ - “I went from having what was a simple sinus infection spread to my chest. I had that for six months. Then I got the flu. Then I got COVID. Then I’m pretty sure I got pneumonia. Yeah, there was a really bad six months, and really scary, because it all presented very differently, very atypically. So doctors really weren't sure what was going on. So that was a reminder you are still immunocompromised. You still have NMO. And like, nine times out of ten. You're fine. But like, hey, you're still sick.” |
| Getting Diagnosed | - “It was three years [between symptom onset and diagnosis]. It took a long time.” - “The first time I got sick, I ended up being in the hospital for 5 weeks, 3 different hospitals, ‘cause I was sick for like an entire month, and everybody kept sending me home. They kept [saying] we don’t see anything, we don’t see anything, and sending me home. It started in February 2012, and I didn’t get it officially rightly diagnosed until February 2015.” |
| Medical Trauma | - “One thing that is so critical in a patient’s life is that moment when you first heard this disease name for the first time. What did that mean for you? I remember it so vividly, my reaction to it, and what thoughts went in my head. My immediate question was, am I going to die from this? I was kind of in like survival mode. It was a fight-or-flight moment. What do I do?” - “The word blind or attack would like lead to [a panic attack] for some reason.” - “[Going to] the dentist, the feeling of being numbed triggers PTSD in me because that was one of my main symptoms, being like, my whole body was numb. And that was one of the hardest things to get over mentally… I just don’t ever like being numb because it reminds me of those times. So I really spiraled when I found out that I had to get a root canal. And I got it, and it’s like fine now, but it was like, leading up to it was awful.” |

Caregiver Interview Qualitative Data

| Loved One’s Pain/Physical Impairment | - “I had, I had to lose myself with my loved one under so much pain. It was killing me. So, I had to lose myself in the science of it.” - “I mean it got stressful just like making sure that she was okay. She was in a lot of pain.” - “The muscle spasms… can be debilitating and painful to watch while it is happening.” |
| --- | --- |
| Loved One’s Vision Impairment | - “[The hardest symptom] would be her blindness. It would definitely be her blindness.” - “In a crowded situation, it can be really chaotic to have my shopping cart, her and the dog all walking down an aisle together. And, you know, if there’s 9 people in the row, it’s 9 people that we have to figure out how to dodge. So if we can condense that down into, uh, it makes shopping easier. Yeah, that’s been one of the hardest little things. You kind of forget, sometimes it just kind of slips your mind that she’s blind.” |
| Loved One’s Emotional Health | - “His life got ruined because of NMO… His life would be way different if this didn’t happen. He was 22 [when he was diagnosed]. He gets the rare disease, and he shouldn’t even have gotten it. I should have gotten it. The indignities he suffered at 22…The nurses were 22, and don’t get me wrong, they were professional, but in any other circumstance he’d be dating those nurses, and instead, they’re changing him because he has no control from the belly button down.” - “If it weren't for NMO, he wouldn't have developed this addiction in the first place for the pain.” - “He had, like, profound trauma from his experiences in the hospital with blindness and paralysis. He called me in December of 2014 and had a lot of dark, depressing thoughts like suicidal ideation.” - “The psychosocial burden, like experiencing trauma of like your body fucking you over like experiencing tremendous pain that, like you, at least at that time can't accept or cope with.” |
| Invisible Illness | - “People who we don't know look at her and think she's normal. And people who we do know who have seen her when it was like she was really hitting the fan, and then now they see her, and they think she's like completely fine. But uh, I mean I spend like twenty-four hours with her. So there's definitely a period where she still struggles.” - Yeah, it’s just nobody knows what it is. Everybody, I have to tell them it’s related to MS. - “[Friends and family] don't see how much pain he is in some days due to his spasms, headaches, or etc. He tries to put up a good front for family.” - “Everyone's like as long as you're not in the hospital, you don't have, like, cancer like, you're fine.” |
| Employment/Finances | - “You know, we had to downsize our home, but it was, still, a team situation.” - “I worry that he won't be able to work any longer. Right now he is our sole provider. If something were to happen where he was unable to work, I will have to go back into the work field.” - “When you don't work, then people don't think you have anything to contribute right or like the indignities you suffer because you're not part of belonging because you don't go up to work.” |
| Anxiety | - “I think there's definitely a lingering like everyday kind of fear that like she might relapse.” - “I just worry about her when I’m gone, you know. And knowing what her situation will be, you know. If it stays the way it is, and that she kind of, on her own. Because, I think, nobody steps up like a mother.” - “I’m scared to wonder what's going to happen in the next five years? Is she going to get worse? Um, you know. How is this going to progress?” - “I became like obsessed with NMO. From a science perspective.” |
| Depression | - “[NMOSD] doesn't make me afraid. It just, you know, it just um puts the damper on things.” - “So you find yourself going through all the stages of grief with a lot of denial and bargaining.” |
| Social Life | - “I don't think any of our friends really necessarily had an issue or anything. there's a certain lack of uh empathy that other twenty-year-olds have to a debilitating illness like that. So I think that was probably the biggest thing.” - “I think people think because she’s in a wheelchair, they don’t respect the person.” - “ I just need people to know that [NMOSD] is a part of my life. It's always going to be a part of my life, and they need to understand, like the intensity of that. |
| Perspective of Being a “Caregiver” | - “I think I’m proud that I am able to help her and I’m still on this planet to live, and to uh, uh, exchange the good times with her, you know.” - “Sometimes she's like a little sick kid, and she's like, ‘Oh, I need this’ and she's like asked for stuff every five seconds. And yeah, it's a little... It's a little daunting. You know it, it makes you become a little understanding.” |
| COVID/The Pandemic | - “Now that it’s, a lot of these [rules] are lifted, we still wear a mask. I wear my mask. Anywhere we go in a store, we wear a mask. Outside, we don’t. But if we’re around people, we wear a mask. Why take a chance?” |
| Relationship with Patient | - “We were newlyweds when [she was diagnosed with NMOSD], we were only married for two years. So, this was…you know, there was more than once when she was like, ‘I know you didn’t sign up for this, I wouldn’t be…it’s okay if you want to go with somebody or just go and have a normal life’ and I’d go, I actually did sign up for this, it’s called in sickness and in health. I’m not going anywhere unless you file a restraining order.” - “I feel like a lot of times I'm like an extension of her. She's like an extension of me kind of thing. We have spent a lot of time together. I think [NMOSD] has brought us closer.” - “When she was so sick and couldn’t go anywhere, I used to just put her in the car and drive her by McDonalds, all the windows down, and we had a picnic in the car. We did that several times, and we had to laugh, because it was our picnic. I love the picnic in the car.” - “Sometimes I get frustrated with him when I don't mean to be. Like when he's trying to do something and I know he is hurting but he won't go sit down and let me handle it. He gets mad because he wants to be able to do stuff to help me out.” - “Our intimacy time isn't the same before he was diagnosed.” |
| Caregiving Responsibilities | - “I’m the driver. Um, you know, it’s…I don’t really even look at it as caregiving responsibilities, it’s just teammate responsibilities and husband responsibilities. And, um, I don’t think separate the two. I mean I do 90% of the cooking, she only cooks when she wants to. It’s…she decides she wants to do something, so I usually will prep everything ahead of time, and then set them up in order in which they need to be used, on the cutting board, next to the stove. And all she needs to do is put it all together. So, it’s about teamwork” - “It felt more…less like a husband and more like a caregiver” - “I try to not stress her out.” - “Well, I come over every day and like this weekend, we spend in my house. I’m only 3 miles away and a phone call away if anything ever happens.” - “I told her, I’m your uber because I take you to appointments, and she has quite a few. I mean if it’s not infusion appointment, it’s a specialty doctor for something else.” - “I drive him everywhere for doctor appointments and work. I do yard work since he is unable to be outside in the heat. It has gotten a little bit easier since my teenagers are able to help out more around the house.” - “On his bad days, I have to help him get into the house if his legs are not working well, or help him get into bed because his legs are heavy and won't listen.” |
