# Supplementary material for: The psychological burden of NMOSD – a mixed method study of patients and caregivers
Source: PLoS One. 2024 Mar 29;19(3):e0300777. doi: 10.1371/journal.pone.0300777 (PMC10980246; doi:10.1371/journal.pone.0300777)
Supplement: Attachment — Submitted filename: The review.docx [file pone.0300777.s004.docx]

**The review**

This study aims to describe and understand the psychological burden of patients with NMOSD and their caregiver/loved ones via self-report measures, focus groups, and in-depth interviews.

This study aims to identify and categorize salient features of the psychological burden specific to NMOSD to best inform effective intervention approaches.

Describing and understanding the psychological burden of patients with NMOSD and their caregivers/loved ones can be helpful in use of bio-psychosocial approach to NMOSD. The claims are properly placed in the context of the previous literature. The authors treated the literature fairly.

However, following are minor and major comments.

**Minor comments**

The text must be re-read and corrected, there are some problems and I give some examples.

1. Page 16; “Many patients and caregivers shared about long timeframes”. Clarify how many patients and caregivers in the total sample.

2. Page 18; “Many patients and caregivers reported significant emotional distress immediately following diagnosis of NMOSD, including isolation as a person with a rare disease and a near total lack of information provided by medical professionals”. Are: “isolation as a person with a rare disease and a near total lack of information provided by medical professionals”, components of emotional distress or these can lead to emotional distress?

3. Page 24; “While most patients reported some level of depressive symptoms, including depression specifically related to life with NMSD, about a third endorsed clinically relevant depression. What this does mean?

4. Page 24; “(...) most patients and half (...). You can clarify.

**Major comments**

1. For the psychometrics measures, reliabilities would have been assessed using at least alpha of Cronbach.

2. Analyses seem not performed to a high technical standard.

2.1. When we have a small sample (N=30), the use of percentages can be questionable. For example, if n= 4 what does mean 13.3% of this n? (see page 11-12,...), this seems not appropriate.

2.2. Before computing the bivariate analysis, was the normality testing performed? If not, the estimation of values for parametric tests is not possible but they are estimation techniques other than normal theory, such as bootstrapping, an alluring estimation technique that requires few assumptions and deals with non-normally distributed data, this last could have been used.

3. To decide that participants had significant symptoms of depression, anxiety, trauma, cognitive fusion (,...) what were criteria (for example clinical symptoms could have been identified based on cut-off scores).
