# Supplementary material for: The psychological burden of NMOSD – a mixed method study of patients and caregivers
Source: PLoS One. 2024 Mar 29;19(3):e0300777. doi: 10.1371/journal.pone.0300777 (PMC10980246; doi:10.1371/journal.pone.0300777)
Supplement: Attachment — Submitted filename: Comments.docx [file pone.0300777.s005.docx]

**EVALUATION**

Thank you for allowing me to read this revised manuscript. Having reread the manuscript, the authors have addressed most of my comments. However, there are a few comments to address before heading to another step.

**Page 4-5**: Generally, paragraphs are five to ten lines in length. A paragraph of two to four lines doesn’t look smart.

**Pages 7-8**: Socio-demographic characteristics of the participants should be removed from the materials and methods section to be pasted and analyzed in the results section.

**Pages 14-18**: Information regarding descriptive analysis of study variables and the rate of psychological burden was clearly mentioned in the manuscript. However, there are no tables summarizing this information.

Also, can you provide information on other measurements of variability such as standard deviation, skewness, and kurtosis? Descriptive analysis of study variables based only on the mean doesn’t provide much information.

**Pages 22-25**: It seems that your conclusion section reflects the discussion section. The reason is that you have explained what you found, showing how it correlates with the existing literature, which makes it a discussion section. Consequently, you can then set up a conclusion section apart to summarize your findings and explain why they are very important. Then, I suggest the following sections: Results, Discussion, Conclusions,…
