# Supplementary material for: The psychological burden of NMOSD – a mixed method study of patients and caregivers
Source: PLoS One. 2024 Mar 29;19(3):e0300777. doi: 10.1371/journal.pone.0300777 (PMC10980246; doi:10.1371/journal.pone.0300777)
Supplement: Attachment — Submitted filename: Response to Reviewers 2nd revision.docx [file pone.0300777.s006.docx]

Teresa Juárez-Cedillo, PhD
Academic Editor
PLOS ONE

Dear Dr. Juárez-Cedillo,

Thank you for the detailed feedback regarding our manuscript, “The psychological burden of NMOSD – a mixed method study of patients and caregivers” (PONE-D-23-06896R1). We are aware of the considerable amount of time and effort that go into reviewing a manuscript, and we are very appreciative of the attention to our work and the thoughtful suggestions made.

Below are our responses to the reviewer comments. We are confident that a more concise and focused manuscript results from the reviewer’s thoughtful feedback.

**ACADEMIC EDITOR:**

**Comment 1:** Reviewing the length of the introduction focusing on considering the most relevant elements reported in the literature.

**RESPONSE TO COMMENT #1:** Thank you – we have condensed the introduction considerably, focusing only on

**Comment 2:** All data analyzed from the study population must be in the results section.

**RESPONSE TO COMMENT #2:** Yes – these are now all included in the results section.

**Comment 3**: Combine the conclusion with the discussion and write a new conclusion with the most relevant findings of this study.

**RESPONSE TO COMMENT #3:** Thank you – we have created a new Conclusions and Future Directions section to address this feedback.

**Reviewer #1:**

**Comment 1:** The front component/introduction is quite lengthy and difficult to get through. I'm not sure there is a need to go through paragraphs of each individual construct and the existing literature (e.g. coping, psychological resilience). I feel that it is more important to synthesise the literature to highlight the gaps in knowledge.

**RESPONSE TO COMMENT #1:** Agree – we have condensed the intro considerably.

**Reviewer #2:**

**Comment 1:** Page 4-5: Generally, paragraphs are five to ten lines in length. A paragraph of two to four lines doesn’t look smart.

**RESPONSE TO COMMENT #1:** Agree – we have consolidated paragraphs in the newly revised and condensed introduction.

**Comment 2**: Pages 7-8: Socio-demographic characteristics of the participants should be removed from the materials and methods section to be pasted and analyzed in the results section.

**RESPONSE TO COMMENT #2:** Thank you for this correction, we have moved this information and associated Tables 1 and 2 accordingly, to the results section.

**Comment 3:** Pages 14-18: Information regarding descriptive analysis of study variables and the rate of psychological burden was clearly mentioned in the manuscript. However, there are no tables summarizing this information.

**RESPONSE TO COMMENT #3:** Thank you – we have created such a table summarizing the descriptive analysis of study variables. This is now Table 3.

**Comment 4:** Also, can you provide information on other measurements of variability such as standard deviation, skewness, and kurtosis? Descriptive analysis of study variables based only on the mean doesn’t provide much information.

**RESPONSE TO COMMENT #4:** Yes, agree - we have included these measures of variability (standard deviation, skewness and kurtosis) in our new Table 3.

**Comment 5:** Pages 22-25: It seems that your conclusion section reflects the discussion section. The reason is that you have explained what you found, showing how it correlates with the existing literature, which makes it a discussion section. Consequently, you can then set up a conclusion section apart to summarize your findings and explain why they are very important. Then, I suggest the following sections: Results, Discussion, Conclusions,…

**RESPONSE TO COMMENT #5:** Thank you for this suggestion. We have now differentiated Results from Discussion sections and, rather than creating much additional text, we have created an integrated Conclusions and Future Directions section.

We have electronically submitted the revised manuscript. We hope that we have addressed all comments and suggestions appropriately. If not, we will be happy to consider making whatever additional changes you or the reviewers request. Thank you for continuing to consider this manuscript for publication in *PLOS ONE.* We look forward to hearing from you.

Sincerely,

C. Virginia O’Hayer, Ph.D.

Clinical Professor

Director, Jefferson Center City Clinic for Behavioral Medicine

Department of Psychiatry & Human Behavior

Thomas Jefferson University Hospital
